# Whole-Genome Analysis of *Mycobacterium avium* subsp. *paratuberculosis* IS*900* **Insertions Reveals Strain Type**-**Specific Modalities**

**DOI:** 10.3389/fmicb.2021.660002

**Published:** 2021-05-10

**Authors:** Cyril Conde, Marian Price-Carter, Thierry Cochard, Maxime Branger, Karen Stevenson, Richard Whittington, John P. Bannantine, Franck Biet

**Affiliations:** ^1^INRAE, ISP, Université de Tours, Nouzilly, France; ^2^AgResearch Ltd., Hopkirk Research Institute, Palmerston North, New Zealand; ^3^Moredun Research Institute, Penicuik, United Kingdom; ^4^School of Veterinary Science, The University of Sydney, Sydney, NSW, Australia; ^5^USDA-ARS, National Animal Disease Center, Ames, IA, United States

**Keywords:** *Mycobacterium avium* subsp. *paratuberculosis*, insertion sequence IS*900*, evolution, RFLP, complete genome

## Abstract

*Mycobacterium avium* subsp. *paratuberculosis* (*Map*) is the etiological agent of Johne’s disease in ruminants. The IS*900* insertion sequence (IS) has been used widely as an epidemiological marker and target for PCR diagnosis. Updated DNA sequencing technologies have led to a rapid increase in available *Map* genomes, which makes it possible to analyze the distribution of IS*900* in this slow-growing bacterium. The objective of this study is to characterize the distribution of the IS*900* element and how it affects genomic evolution and gene function of *Map*. A secondary goal is to develop automated *in silico* restriction fragment length polymorphism (RFLP) analysis using IS*900*. Complete genomes from the major phylogenetic lineages known as C-type and S-type (including subtypes I and III), were chosen to represent the genetic diversity of *Map*. IS*900* elements were located in these genomes using BLAST software and the relevant fragments extracted. An *in silico* RFLP analysis using the *Bst*EII restriction site was performed to obtain exact sizes of the DNA fragments carrying a copy of IS*900* and the resulting RFLP profiles were analyzed and compared by digital visualization of the separated restriction fragments. The program developed for this study allowed automated localization of IS*900* sequences to identify their position within each genome along with the exact number of copies per genome. The number of IS*900* copies ranged from 16 in the C-type isolate to 22 in the S-type subtype I isolate. A loci-by-loci sequence alignment of all IS*900* copies within the three genomes revealed new sequence polymorphisms that define three sequevars distinguishing the subtypes. Nine IS*900* insertion site locations were conserved across all genomes studied while smaller subsets were unique to a particular lineage. Preferential insertion motif sequences were identified for IS*900* along with genes bordering all IS*900* insertions. Rarely did IS*900* insert within coding sequences as only three genes were disrupted in this way. This study makes it possible to automate IS*900* distribution in *Map* genomes to enrich knowledge on the distribution dynamics of this IS for epidemiological purposes, for understanding *Map* evolution and for studying the biological implications of IS*900* insertions.

## Introduction

Among mycobacteria that cause severe disease in animals, *Mycobacterium avium* subspecies *paratuberculosis* (*Map*) occupies a leading position in terms of economic importance and welfare effects on livestock worldwide. Paratuberculosis or Johne’s disease has been recognized as a major disease of ruminants since the late nineteenth century and continues to spread in most industrialized countries (Rathnaiah et al., 2017). This disease has a significant economic impact on livestock on all continents despite expensive eradication programs existing worldwide and the use of vaccines. Today, the prophylaxis against paratuberculosis is costly and inefficient leading to great concern for controlling this endemic disease (Barkema et al., 2010; Whittington et al., 2019).

Despite the publication of the complete genome sequence of *Map*, knowledge of the biology and pathogenesis of this bacterium remains largely unclear. Genomic studies have revealed that *Map* evolved from *Mycobacterium avium* subsp. *hominissuis* (*Mah*) into two distinct lineages (Turenne et al., 2008). These lineages are historically referred to as the cattle (C) -type and the sheep (S) -type and can be consistently distinguished by the genotyping tools available for *Map* including VNTR, SSR, and SNP analysis (Bannantine et al., 2013). In addition to their genomic diversity, strains belonging to these two lineages exhibit phenotypic differences (Taylor, 1951; Stevenson et al., 2002; Janagama et al., 2009; Lefrancois et al., 2013; Bannantine et al., 2017). In general, strains belonging to the S lineage are more common in sheep and goats whereas those from the C lineage predominate in cattle but can be isolated from a wider host range including deer, bison and humans. The association of each lineage with either cattle or sheep hosts is not exclusive since strains representative of either lineage can cause disease in all types of ruminants (Stevenson, 2015). More recently, whole genome studies have revealed subdivision of the S lineage into two subtypes I and III, and confirm that both are distinct from the C-type (Bryant et al., 2016).

The complete, annotated genome sequence, of isolate *Map* K-10 of C-type was published in 2005 (Li et al., 2005). Very recently the complete genome of S-type *Map* including the genome of the Telford strain subtype I and the strains S397 and JIII-386 of subtype III have been established (Brauning et al., 2019; Wibberg et al., 2020; Bannantine et al., 2021). Recent studies have shown that C-type strains of *Map* have a very homogenous genome, but the S-type I strain Telford has a different genomic organization and more single nucleotide polymorphisms (SNPs) compared to the C-type *Map* strains (Uchiya et al., 2017; Bannantine et al., 2020; Wibberg et al., 2020). Furthermore, *Map* genomes aligned at a common start revealed that C-type strains have a highly homologous genome synteny while the S-type strains (both subtypes I and III) show large rearrangements (Bannantine et al., 2020; Wibberg et al., 2020).

Mobile genetic elements play a key role in chromosomal remodeling and host adaptation (Moran and Plague, 2004; Toft and Andersson, 2010). Among the insertion sequences (IS) present in the *Map* genome, the IS*900* element is exclusively found in this subspecies (Green et al., 1989; Bull et al., 2000; Semret et al., 2006). This element varies from 16 to 22 copies per genome. These features make IS*900* a robust marker for the diagnosis of *Map*. Moreover, this sequence has long been used for the study of the polymorphism of *Map* strains. IS*900* is highly conserved across the two distinctive S and C lineages (Semret et al., 2006). The phylogeny based on IS*900*-RFLP distinguishes both these lineages and the subtypes I and III of *Map* (Pavlik et al., 1999; Biet et al., 2012). Unfortunately, this technique is time consuming, requiring a large amount of DNA and therefore dependent on very tedious culture of *Map* (Choy et al., 1998; Whittington et al., 2000). Consequently, this method is under-utilized or even abandoned. As a result, we developed a program to produce *in silico* IS*900* RFLP patterns based on complete genome sequences to simplify the characterization of newly sequenced isolates.

IS*900* belongs to the IS*110* family of insertion sequences because they do not contain the typical terminal inverted repeat sequences and do not generate flanking direct target DNA repeats on insertion. The sequence of IS*900* was first deposited under accession number X1629 (Green et al., 1989). The size of IS*900* is 1,451 base pairs (bp). The sequence of IS*900* contains a 1,200 bp gene encoding a putative transposase termed P43 belonging to the DDED family of transposases (Tizard et al., 1992), which contain a characteristic motif of three catalytic residues, two of which are aspartic acids and a third position that is either glutamic or aspartic acid (Nesmelova and Hackett, 2010). The sequence of IS*900* also contains a second ORF, encoded on the complementary strand to the transposase, designated the *hed* gene (host expression dependent) of unknown function (Doran et al., 1997). This ORF spans the entire IS*900* sequence but it does not contain a putative RBS or start codon (Supplementary Figure 3). Using a non-replicating vector, England et al. (1991) showed that integration of IS*900* can transpose by simple insertion as well as by a replicative mechanism. The biological implications of IS*900* transposition during *Map* adaptation to ruminant hosts and its evolution into two distinct lineages related to host specificity remain unknown.

In this study, we analyzed the chromosomal distribution of IS*900* across the primary *Map* C and S lineages and identified IS*900* sequence polymorphisms characteristic of each lineage and S-type subtypes. A bioinformatic RFLP genotyping method was developed to characterize the complete genomes of *Map* now available as well as those sequenced in the future. This program enables RFLP analysis *in silico*, including digital visualization for phylogenetic purposes. Finally, the distribution and analysis of IS*900* in *Map* has evolutionary implications for this veterinary pathogen.

## Materials and Methods

### Strains and Genomes

Strain K-10 (Li et al., 2005, 2019), Telford (Brauning et al., 2019) and S397 (Bannantine et al., 2012) were included in this study as references of the major lineages that have emerged during the evolution of *Map*. Isolates were propagated on slopes of modified Middlebrook 7H11 supplemented with 20% (vol/vol) heat inactivated newborn calf serum, 2.5% (vol/vol) glycerol, 2 mM asparagine, 10% (vol/vol) Middlebrook oleic acid-albumin- dextrose-catalase (OADC) enrichment medium (Becton Dickinson, Oxford, Oxfordshire, United Kingdom), Selectatabs (code MS 24; MAST Laboratories Ltd., Merseyside, United Kingdom), and 2 μg ml-1 mycobactin J (Allied Monitor, Fayette, MO, United States). The complete genome sequence of K-10 (C-type, NC_002944.2), Telford (S-type subtype I, NZ_CP033688.1) and S397 (S-type subtype III, NZ_CP053749.1) were downloaded from the NCBI RefSeq (O’Leary et al., 2016; Table 1). S397 was annotated using PGAP (Tatusova et al., 2016).

**TABLE 1:** Details of the strains and genomes used and information on the number of copies of the IS*900*.

**Strain**	**Telford**	**K-10**	**S397**
Type	S	C	S
Subtype	I	II	III
IS*900 Bst*EII RFLP profile	S1	R01	A
Number of bands detected *in vitro*	17	15	15
Number of bands detected *in silico*	22	17	19
Number of contigs	1	1	1
Accession number	NZ_CP033688.1	NC_002944.2	NZ_CP053749.1

### *In vitro* IS*900*-RFLP

*Mycobacterium avium* subsp. *paratuberculosis* strains were typed by *Bst*EII IS*900*-RFLP as described previously (Thibault et al., 2007). Profiles were designated according to nomenclature previously described (Collins et al., 1997; Pavlik et al., 1999; Mobius et al., 2009). Profiles were analyzed using Bionumerics^TM^ software version 7.6.3 (Applied Maths, Belgium).

### Bioinformatic Analysis

#### IS*900* Sequence Identification and *in silico* IS*900* RFLP Workflow

**FIGURE 1:**
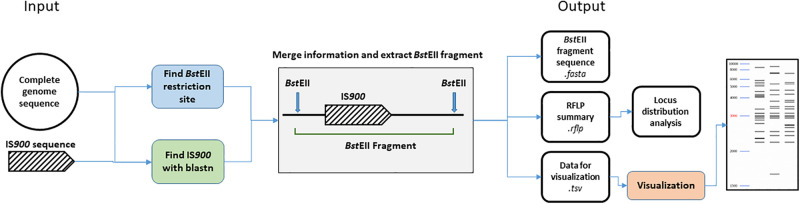
Bioinformatic analysis pipeline. Details of steps performed by the IS*900* RFLP *in silico* pipeline. From complete genome sequences, *Bst*EII restriction site and IS*900* sequence positions were identified. Both data sets are merged together to extract the *Bst*EII fragment sizes from the genome sequence. Previous positions IS*900* and sequence orientation are stored in a.rflp file for further analysis. *Bst*EII fragment sizes are converted into migration distance based on calculation from an *in vitro* gel migration (see section “Materials and Methods”) and saved in the .tsv file for further visualization of the RFLP profile.

We developed an *in silico* analysis pipeline for IS*900* RFLP profiling using complete genome sequences as the input (Figure 1). As a first step, all *Bst*EII restriction sites were located in the genome using in-house script (available at: https://forgemia.inra.fr/public-pgba/is900-rflp-in-silico) developed with Biopython (v1.76) (Cock et al., 2009). IS*900* copies in the genome sequence were identified using a blastn version 2.9.0 (Altschul et al., 1990) search of IS*900* sequence retrieved from the NCBI database (accession no. X16293) with a percent identity of 99% and an *e*-value of 1e-100 to exclude all false positive hits. For each hit, upstream and downstream sequences nearest the *Bst*EII restriction sites were retrieved from the *Bst*EII restriction map and length of the *Bst*EII fragment was computed. A gel migration equation was previously determined using GelAnalyzer 19.1^1^ and used to convert fragment length into migration distance for further visualization of the RFLP profile. Migration data and coordinates of IS*900* copies were saved in .tsv and .rflp files, respectively, for visualization of the profile and further investigation of locus distribution. Visualization of RFLP profiles was performed using python library matplotlib (v3.3.0)^2^.

#### IS*900* Sequence Polymorphism

In order to confirm IS*900* sequence polymorphisms previously described (Semret et al., 2006; Castellanos et al., 2009), IS*900* copies from the three genomes were extracted and aligned using Multalin (Corpet, 1988) with the “DNA” symbol comparison table. Shorter IS*900* copies in the alignment were manually checked with Artemis software version 18.1.0 (Carver et al., 2008) to confirm blastn results.

#### Mauve Alignment

Synteny alignments were determined with Mauve (snapshot_2015-02-13 build 0) (Darling et al., 2004). In order to avoid false indications of inversions or other rearrangements, these genomes were first shifted to start at the *dnaA* gene prior to Mauve analysis. Using the complete genome sequences of each strain, we performed a 1 vs. 1 genome alignment using progressive Mauve (Darling et al., 2010) and also an alignment of the three genome sequences in order to visualize differences in genomic organization.

#### Orthology Analysis

To identify IS*900* copies inserted at orthologous genomic sites between the genomes of K-10, Telford and S397, we performed blastn searches using as queries, 2,000 bp of upstream and downstream genomic regions flanking each IS*900* copy. These orthologous flanking regions from one genome were aligned to the two other genomes and compared to identify orthologous loci. Therefore, blastn results were parsed in order to select the best match. The best match was defined with the following criteria: (1) an *e*-value of 0 and (2) a minimum coverage of 80%. For many flanking regions, blastn yielded only one result. But for a few others, more than one result was returned. In cases where the coverage of the best result is below 80%, we searched for other results around 4,000 bp from the first result to identify genome rearrangement/differences and merged them. Finally, orthologous loci were considered linked to an IS*900* loci if the center of the BLAST hit fell within the 2,000 bp region flanking the IS*900* element in the target genome.

#### IS*900* Sites Gene Ontology Enrichment Analysis

This approach aimed to determine if the genes near insertion sites were enriched for any particular function. Protein sequences upstream and downstream of IS*900* were extracted, if available, from the RefSeq annotation. Functional annotation was performed using eggNOG-mapper-2.0.1 (Huerta-Cepas et al., 2017) based on eggNOG orthology data (Huerta-Cepas et al., 2019). Sequence searches were performed using DIAMOND version 2.0.5 (Buchfink et al., 2015).

#### Identifying the Candidate Insertion Sites

In order to identify targeted insertion site motifs in the genomes of *Map*, 10 bp of upstream and downstream sequence of each IS*900* were extracted. Each strand of the IS element was considered and extracted sequences were reverse complemented if needed. Insertion sequences containing deletions or duplications were excluded from the analysis. Multalin (Corpet, 1988) was used to align upstream and downstream sequences using the “DNA” symbol comparison table. The alignment of the upstream sequence was performed with “gap penalty at opening” set to 1 and “gap penalty at extension” set to 0. The alignment of the downstream sequence was performed with default parameters. Upstream and downstream IS*900* flanking regions from each genome were aligned to the *M. avium* subsp. *hominissuis* (*Mah*) 104 genome using blastn in order to find orthologous regions. Only adjacent regions have been retained. Putative target sequences, previously identified in the three genomes, were extracted manually from the *Mah* 104 genome using Artemis software version 18.1.0 (Carver et al., 2008) based on blastn results. MEME version 5.3.1 (Bailey and Elkan, 1994) was used to identify a putative target site motif.

## Results

### IS*900* Sequence Identification in Complete *Map* Genomes

**FIGURE 2:**
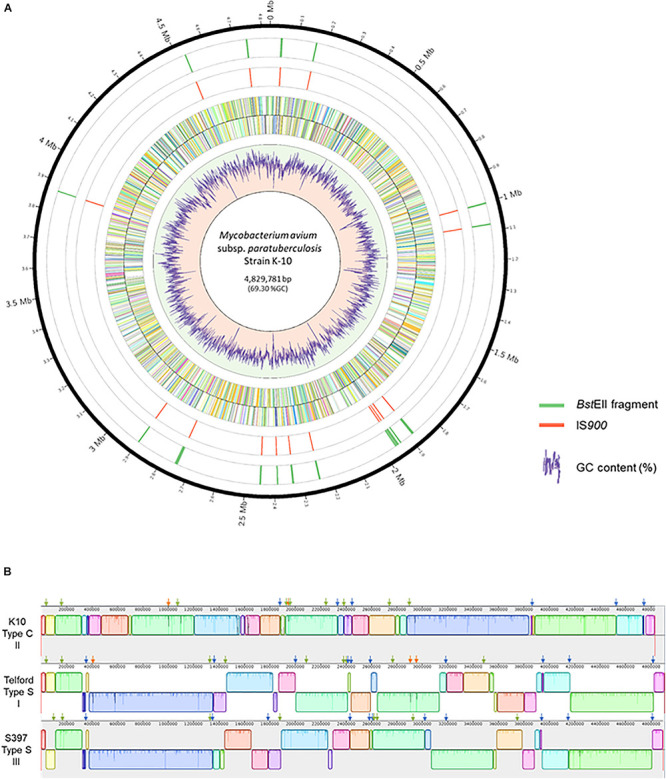
Distribution of IS*900* copies on the genome of *Mycobacterium avium* subsp. *paratuberculosis* strain K-10. **(A)** Shown, using Circos version 0.69–8, from the outer circle to the inner circle are the megabase (Mb) positions on the chromosome, the *Bst*EII restriction sites, the position of each IS*900* element, plus strand ORFs, minus strand ORFs and a plot of the percent GC. **(B)** Mauve alignment of K-10 (top) with Telford (middle) and S397 (bottom) showing genomic reorganization of the genomes. The colored boxes represent homologous regions present in each genome. Blocks below the centerline indicate regions with inverse orientation. Regions outside the blocks lack homology between the genomes. Within each block there is a similarity profile of the DNA sequences and the white areas indicate sequences specific to a genome. The scale is in base pairs. Orthologous insertions are indicated, using the slider of Mauve, by a green arrow, specific insertions are indicated by a orange arrow and conserved loci only in two genomes by a blue arrow.

The complete genome sequences of *Map* strains representing the three known genetic lineages provide a unique opportunity to analyze the distribution of IS*900* across all *Map* strains. The search for IS*900* sequences in complete genomes identified 17 copies in the K-10 genome, 19 copies in the S397 genome and 22 copies in the Telford genome (Table 1). To investigate if the expansion of IS*900* is correlated with the evolutionary scenario of *Map*, additional analysis on 10 available C-type complete genome sequences shows that between 16 and 17 IS*900* copies are consistently observed, which is less than the number of copies identified in S-type strains (Supplementary Table 3). The advantage of having the complete genome is to be able to precisely locate the IS*900* sequences on the chromosome. Figure 2A shows the positions of IS*900* on the circular chromosome of K-10. In parallel with the evolution of *Map*, genomic organization of the three genetic lineages show numerous large rearrangements that impact the distribution of IS*900* copies within these three genomes. This observation is illustrated in Figure 2B, which shows the mauve alignment of K-10, Telford, and S397 genomes along with the position of all IS*900* insertions.

In addition, these three complete genomes were used to ascertain that SNP polymorphisms in the IS*900* sequences are type-specific. To demonstrate this, 58 sequences representing all copies of IS*900* from the three genomes were aligned.

The alignment in Supplementary Figure 1 shows three positions for which we observed SNPs. Remarkably, all 19 copies of the IS*900* sequence in S397 have a SNP at position 169 (C-T). The second polymorphism is at position 216 with SNPs identified only in Telford and S397 but not on all loci. In Telford 19/22 loci show an A-G substitution. In the S397 7/19 loci show this same A-G substitution. Interestingly, this study reports for the first time a third SNP in IS*900*. Indeed, by alignment of sequences at each locus, we detected only in Telford at position 1406 a T-G substitution on eight of the 22 loci. These results were verified on all available complete genomes of *Map* through the alignment of 175 IS*900* sequences. Further genome analysis has revealed other novel features of the IS*900* sequences. Four loci have shorter sequences, loci 11 and 17 in K-10 have deletions of 44 and 70 bp, respectively, at the start of the sequence. Furthermore, loci seven in Telford and S397 has a 44-bp deletion at the start of the sequence (Supplementary Figure 1).

This analysis also identified a sequence repetition of the motif ACCTTTCTTGAAGGGTGTTCGGGG from position six, two times at locus nine in Telford and three times at locus 13 of S397.

To complete this analysis, we examined the other complete *Map* genomes in NCBI of C-type. The blastn result showed only one substitution A/G at position 981 in one of the 16 IS*900* copies in the MAPK-CN7/15 genome and one substitution C/T at position 1,142 in one of the 16 IS*900* copies in the MAP4 genome. This result correlated with the finding described above and confirms the high degree of conservation of the IS*900* sequence, especially in C-type.

### IS*900* Restriction Fragment Length Polymorphism (RFLP) *in silico*

**FIGURE 3:**
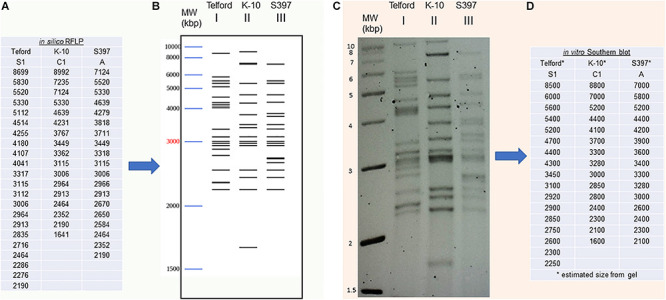
IS*900* restriction fragment length polymorphism (RFLP) “*in vitro*” versus “*in silico*” **(A)** An *in silico* RFLP analysis was developed using complete genomes. This automated procedure identified the *Bst*EII restriction sites to obtain the exact size of all DNA fragments carrying a copy of the IS*900* sequence. IS*900* RFLP profiles were compared using fragment sizes **(A)** or **(B)** by digital visualization of the restriction fragments separated according to their size by mimicking their migration in agarose gel. **(C)** The IS*900* RFLP profiles obtained by classical Southern blot method and hybridization to IS*900* were used to find the approximate fragment sizes by band analysis software **(D)**.

We developed a bioinformatic method that automatically searches the exact positions of IS*900* in each genome. Using this tool, it is now possible to analyze and catalog IS*900* RFLP patterns *in silico*. Building on the IS*900*-RFLP technique used for *Map* strain characterization (Pavlik et al., 1999; Stevenson et al., 2002; Mobius et al., 2009; Biet et al., 2012), our procedure combines the location of IS*900* sequences with the generation of *Bst*EII restriction fragments. The resulting output lists all the fragments with the exact size carrying a copy of IS*900* (Figure 3A). From these data the program provides a digital visualization of the restriction fragments separated according to their size by mimicking their migration pattern in an agarose gel (Figure 3B). For comparison, the IS*900* RFLP profiles obtained by the classical Southern blot method (Figure 3C) were used to find the approximate size and number of *Bst*EII restriction fragments (Figure 3D). As shown in Figures 3B,C, the profiles obtained *in silico* and *in vitro* are highly consistent. More importantly, these profiles deduced from *in silico* genomic analysis can now be compared to those obtained previously by the classical technique.

**FIGURE 4:**
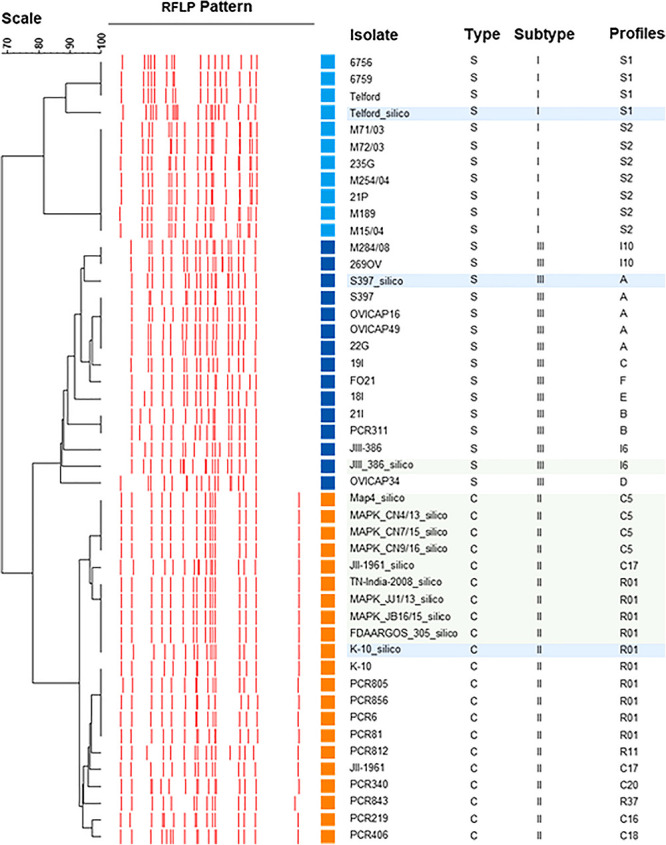
IS*900* RFLP Phylogeny. Phylogeny RFLP with profiles established *in silico* and integrated into the database are indicated by blue boxes for the three reference genomes of C-type and S-type subtypes I and III and in green boxes for the other complete genomes available for *Map*.

The UPGMA dendrogram presented in Figure 4, adapted from Biet et al. (2012), shows an updated phylogeny based on IS*900* RFLP typing where the new *in silico* profiles inferred from K-10, Telford, and S397 IS*900* RFLP analysis have been included together with the *in silico* profiles inferred from the ten recently available genome sequences (Figure 4).

### Abundance, Distribution, and Orthology of IS*900* Copies Between the *Map* Lineages

Next, we sought to identify each site of IS*900* insertion within the three genomes representing the *Map* lineages C and S subtypes I and III. This approach was taken to investigate the role played by these insertions in the genomic evolution of *Map* as well as to identify sites that are enriched for unique insertions that might indicate distinct functionally important genes and pathways. Finally, we analyzed the sequence context of IS*900* insertions to determine their target-site specificity. Using MEME (Bailey and Elkan, 1994) motif analysis software, we identified insertions that have a significant target sequence motif of CATGNNNNNNTCTCCTT (Supplementary Figure 2). The expect values (*E*-value) are small, ranging between 8.6e-46 and 5.5e-66, indicating a high probability that the motif sequence is required for insertion. These alignments are illustrated in Supplementary Figure 2.

**FIGURE 5:**
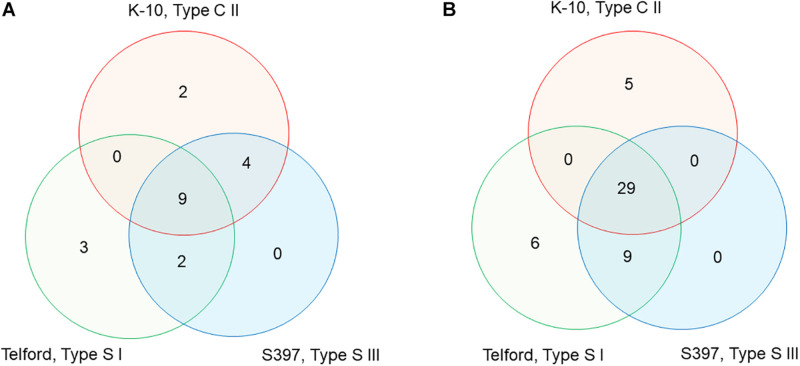
Venn diagram, showing IS*900* orthologous sites and orthologous loci between the three *Map* genomes. **(A)** The diagram indicates the number of IS*900* sites unique or orthologous between 2 and 3 genomes. **(B)** The diagram indicates the number of orthologous or unique loci upstream and downstream of the IS*900* between genomes. Refer to Supplementary Table 2 for additional details.

To characterize the distribution of IS*900* in *Map*, we searched for all genes upstream and downstream from each of the 58 total copies of IS*900* across all three genomes. The directory of all the genes surrounding each copy is presented in Supplementary Tables 1, 2 and Supplementary Figure 3. From this analysis, the IS*900* copies inserted at orthologous genomic sites or absent or polymorphic in each genome were identified. There are nine IS*900* copies inserted in orthologous sites identified across the three genomes, four uniquely shared between K-10 and S397 and two shared between Telford and S397 (Supplementary Table 2 and Figure 5A). The K-10 genome contains two specific sites, Telford genome has three specific sites but no insertions are specific in the S397 genome. For some of the insertion sites, analysis of upstream and downstream genes revealed chromosomal rearrangements. Supplementary Table 2 and Figure 5B indicate the orthologous loci present either upstream or downstream of each IS*900* insertion site. Overall these data show that apart from the three additional IS*900* copies in Telford genome and the 17u locus of K-10, insertion of IS*900* occurred at orthologous sites.

### Effect of IS*900* Insertions in *Map*

**FIGURE 6:**
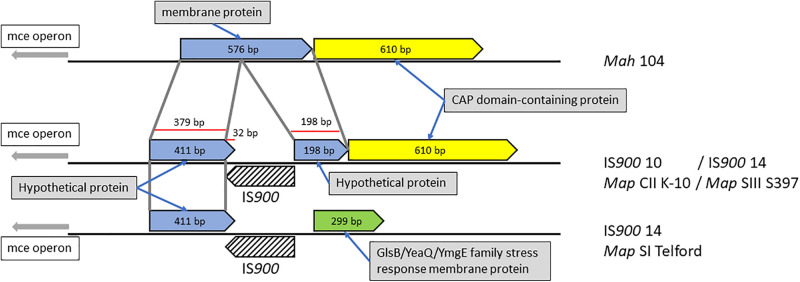
*Mah* gene disrupted by IS*900* insertion in *Map*. Shown are orthologous regions of the genomes of *M. avium* subspecies. The 576 bp *Mah* gene encoding a putative membrane protein is disrupted by insertion of IS*900* into that gene sometime after the subspecies delineation. The corresponding region in the Telford strain appears to have a second genome modifying event, which changed this region upstream of the insertion.

In rare cases, we found that IS*900* insertion sites within predicted coding sequences, indicating loss-of-function for only three disrupted genes. One example is illustrated in Figure 6 and Supplementary Figure 3 where the orthologous loci in *Mah* containing a gene predicted to encode a membrane protein was found disrupted in *Map* by an IS*900* insertion.

**FIGURE 7:**
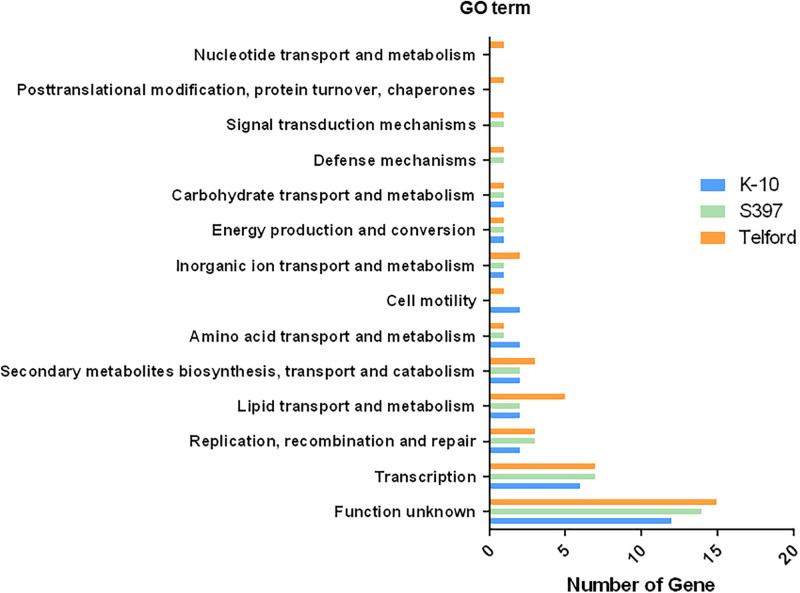
IS*900* sites associated with functional genes and pathways. The diagram indicates Gene Ontology (GO) enrichment analysis of predicted coding sequences near IS*900* sites.

To determine what types of genes are adjacent to IS*900* insertion sites, we performed a Gene Ontology (GO) enrichment analysis of all genes surrounding the IS*900* sites. In this analysis, we identified 13 GO term pathways (Figure 7 and Supplementary Table 3). These analyses found enriched pathways mainly associated with transcription, replication, recombination and repair processes, lipid transport, and metabolism or secondary metabolites biosynthesis, transport, and catabolism.

## Discussion

The *Map* subspecies is an economically significant veterinary pathogen in cattle, sheep and goat populations that is distinguishable from other members of the MAC complex by the presence of IS*900* insertion sequences. It is possible that this transposable element is a driving force in the evolution of *Map*, especially in light of the paucity of genetic recombination and horizontal gene transfer that occurs in this bacterium (Bannantine et al., 2020). In this report we took the unique opportunity to analyze the complete genome sequences of three important *Map* genetic lineages to investigate IS*900* loci-by-loci features and their genomic distribution.

With advances in “long read” sequencing technologies, the resolution of whole genome sequencing (WGS) has improved dramatically. This provides complete genomes required to determine the distribution of insertion sequences. Until WGS became achievable, the only information regarding the presence and number of IS*900* copies were provided by RFLP analysis followed by Southern-blot hybridization to IS*900* (IS*900*-RFLP). Compared to IS*900*-RFLP studies, which require laborious and time-consuming techniques, complete genome analysis gives the exact number and position of each copy of IS*900*.

IS*900* is present in high copy numbers, between 16 and 17 copies in C-type genomes and more in S-type strains (19 copies in subtype III and 22 in subtype I). It is noteworthy that we found 19 copies of IS*900* in the complete genome of strain JIII-386, which was recently published (Wibberg et al., 2020), but this same article reported only 18 copies of IS*900*. However, JIII-386 RFLP data from 2009 does show 19 IS*900* copies (Mobius et al., 2009). This discrepancy is because Wibberg et al. only included intact IS*900* elements, and therefore did not count the IS*900* element at position 106338-107556 in the JIII-386 genome, which is frameshifted by a 2-bp deletion. Analysis extended to the other complete genomes of *Map* confirmed that C-type genomes have a maximum of 17 copies which may be related to the evolutionary history of these genetic lineages (Supplementary Table 3). The example of IS*6110* exclusively found in *M. tuberculosis* Complex (MTBC) has shown that the number of copies of this IS is lineage-specific and classified as either low copy number (*M. bovis*) or high copy number in modern *M. tuberculosis* lineages (Gonzalo-Asensio et al., 2018). However, in *Map* this lineage relationship will require further investigation of additional genomes, especially S-type genomes, to determine if there is any correlation between copy number and host lineage. What is known currently is there are consistently more IS*900* copies in S-type genomes compared to C-type genomes.

The development of RFLP *in silico* analysis provides a useful new tool to automatically define new strain profiles with a resolution that *in vitro* RFLP cannot provide for fragments of close size. These patterns can be compared with the many profiles described in the literature (Pavlik et al., 1999; Whittington et al., 2000; Stevenson et al., 2002; Biet et al., 2012) and in the dedicated *Map* typing application http://mac-inmv.tours.inra.fr (Cochard et al., 2020). The comparison of profiles with the *in silico* approach is facilitated by a numerical comparison of the sizes of the fragments and the knowledge of the associated loci. The *in silico* approach also offers the possibility to choose other restriction sites used for RFLP such as RFLP based on *Pst*I or *Pvu*II sites that have been used in the past (Pavlik et al., 1999; Stevenson et al., 2002; Mobius et al., 2009). The primary limitation of this analysis is the requirement of complete genome sequences. Although sequencing technologies have vastly improved the speed and cost of whole genome sequencing, this still must be acknowledged as a major hurdle to full adaptation of this *in silico* method. Nonetheless, accurate *in silico* RFLP profiles will continue to be developed with each new genome sequence and this will facilitate direct comparisons to newly isolated strains that are not sequenced but are only analyzed by traditional RFLP Southern blot.

This study, using the complete genomes, provided an opportunity to ascertain the degree of conservation of the IS*900* sequence across the different *Map* types. Previous reports, performed on the basis of PCR fragment sequencing, had revealed the existence of type-specific SNP polymorphisms (Semret et al., 2006; Castellanos et al., 2009). Here using the alignment of the sequences of all the IS*900* sites on the three genomes, i.e., 58 individual copies, we have confirmed and clarified the distribution of these SNPs. We have verified that for position 169, the 19 copies of the subtype III strain have a C-T mutation which distinguishes it from subtype I and C-type, which is congruent with the report of Castellanos et al. (2009). Position 216 also exhibits A-G polymorphism in the genome of subtype I and III strains, but only at certain loci. This explains why in previous analysis by sequencing of IS*900* PCR fragments, the results were ambiguous. This report reveals for the first time the existence of a new polymorphic site at position 1406 (T-G), which is only present on eight loci subtype I. Altogether these results show that the sequence of IS*900* remains extremely conserved and that the SNP polymorphism can define three sequevars distinguishing between S- and C-type and even subtypes I and III. These SNPs could be associated with the kinetics of IS*900* expansion in *Map*.

In addition to knowing the distribution of IS*900* copies present in each *Map* genome, we identified and analyzed their insertion site, initially partially studied (Bull et al., 2000), in order to study the expansion features of this IS and to investigate whether IS*900* could play a role in the genomic evolution of *Map*. This report showed that the distribution of IS*900* mainly concerns orthologous loci despite a non-conserved synteny for the genomes of the three types (Bannantine et al., 2020; Wibberg et al., 2020). Interestingly, the S397 genome does not have a specific IS*900* locus, unlike K-10 or Telford, which have two and three specific loci, respectively. Although these results have been confirmed on all complete *Map* genomes available (including 175 sequences of IS*900* aligned see Supplementary Table 3), these analyses could easily be extended to all future *Map* genome sequences.

The study of the genes surrounding each copy of IS*900* was undertaken to determine if the sites impacted might lead to complete loss or modulation of functional genes and pathways. According to our analysis, the insertion of IS*900* was stealthy and did not have a significant impact on gene function, since only a total of three disrupted genes were identified. Interestingly, 51 IS*900* insertion sites are outside coding sequences where the consequences of insertions are more difficult to predict. The GO analysis was performed to determine what type of genes are adjacent to IS*900* sites. Besides the large function unknown GO category, results suggest that insertions most frequently occurred in transcription, replication, recombination and repair processes, lipid transport and metabolism or secondary metabolites biosynthesis, transport and catabolism. The enriched pathways associated with lipid transport might modulate the cell wall biosynthesis, which is a particularity in *Map* and type-specific by production of various lipopeptides exposed in outer membrane of the cell wall of *Map* (Bannantine et al., 2017).

This study shows that the distribution of IS*900* has the potential to provide new insight into *Map* genome evolution, which is linked to the phylogenomic data that distinguishes sheep and cattle lineages and the subtypes. Our observations raise many questions, on the dynamics of transposition of IS*900*, about the significance of the abundance of this IS, a fossil record of *Map* evolutionary dynamics combined with periods of intense transpositional activity. Is there a positive correlation between copy number and host adaptation? Could it be possible that *Map* has maintained copies of this IS to enhance its ability to infect its hosts via a similar evolution strategy to that employed by IS*6110* in TB complex bacteria (Aravin et al., 2007)? Does IS*900* somehow contribute to mycobactin dependency for *in vitro* growth? For all these questions, whole genome sequencing opens up many perspectives on our understanding of the role of IS*900* on the particularities of this ruminant pathogen.

## Data Availability Statement

The datasets presented in this study can be found in online repositories. The names of the repository/repositories and accession number(s) can be found below: https://www.ncbi.nlm.nih.gov/genbank/, NZ_CP033688.1; https://www.ncbi.nlm.nih.gov/genbank/, NC_002944.2; and https://www.ncbi.nlm.nih.gov/genbank/, NZ_CP053749.1.

## Author Contributions

FB and JB conceived and designed the study. All authors made substantial contributions to the analysis and writing of the manuscript.

## Conflict of Interest

The authors declare that the research was conducted in the absence of any commercial or financial relationships that could be construed as a potential conflict of interest.

## Footnotes

1 www.gelanalyzer.com

2 https://ieeexplore.ieee.org/document/4160265

## Supplementary Material

Supplementary Figure 1 IS*900*, highly conserved sequence across all three lineages. Alignment of the IS*900* sequences of the 58 loci identified in the three genomes K10, Telford and S397. On the left is the name of the genome and the number of each loci of the IS*900*. SNP positions are indicated by a yellow arrow and blue or black letters.

Supplementary Figure 2 MEME motif of putative IS*900* insertion site extract from *Mah* 104 genome. Upstream and downstream region extracted from K-10, S397, and Telford genomes were aligned to the *Mah* 104 genome to find orthologous loci. Sequence of 14 to 17 bp present at conserved orthologous loci in the *Mah* 104 genome were extracted and MEME was used to discover conserved motif.

Supplementary Figure 3 Detail of the genes surrounding all the IS*900* loci. Representation of IS*900* loci environment. Gene present 7500-bp upstream and downstream of each IS*900* copies were considered. Gray box represents blastn IS*900* hits. Presence of *hed* gene was indicated when a RBS was found upstream the gene (symbolized by a chevron). The first genes upstream and downstream IS*900* copies were color filled according to their COG functional categories. Pseudo genes were indicated in black.

Supplementary Table 1 Detail of all orthologous loci.

Supplementary Table 2 IS*900* orthologous sites and orthologous loci between the three *Map* genomes.

Supplementary Table 3 IS*900* orthologous analysis across all complete genomes.
