# Supplementary figures and images for: Whole-Genome Analysis of Mycobacterium avium subsp. paratuberculosis IS900 Insertions Reveals Strain Type-Specific Modalities
Source: Front Microbiol. 2021 May 10;12:660002. doi: 10.3389/fmicb.2021.660002 (PMC8141618; doi:10.3389/fmicb.2021.660002)

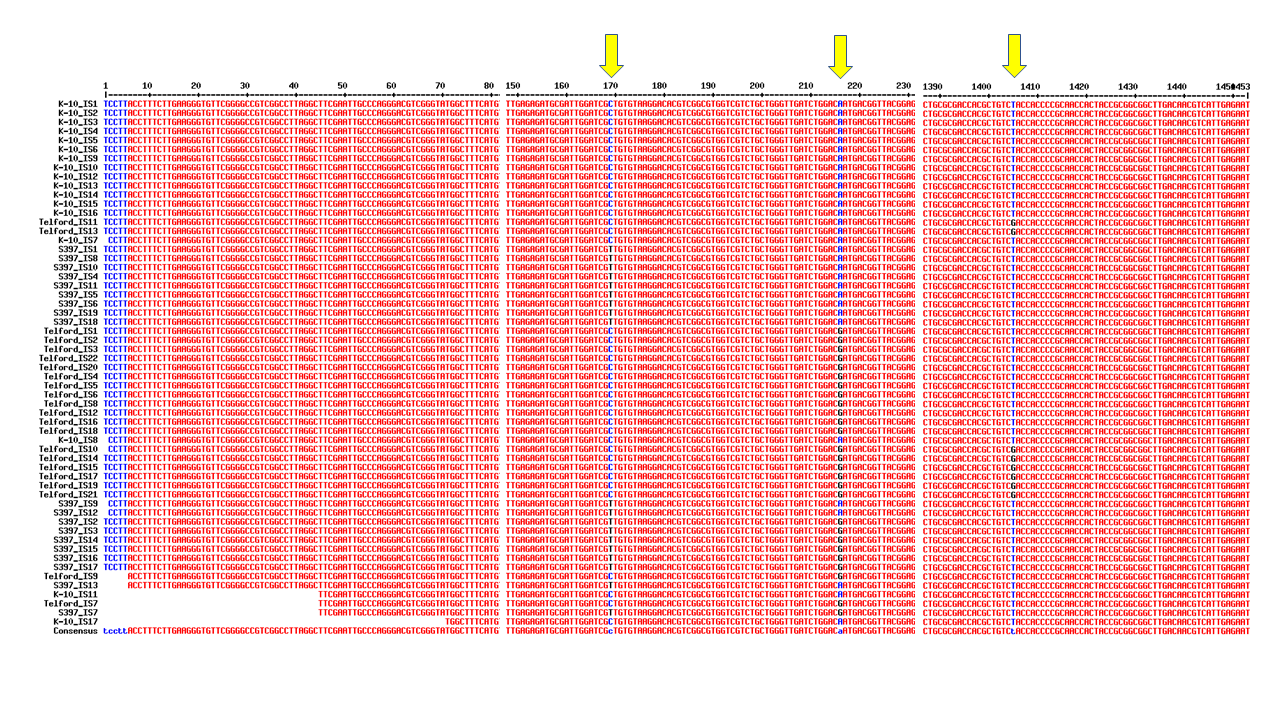

Supplement: Supplementary Figure 1 — IS900, highly conserved sequence across all three lineages. Alignment of the IS900 sequences of the 58 loci identified in the three genomes K10, Telford and S397. On the left is the name of the genome and the number of each loci of the IS900. SNP positions are indicated by a yellow arrow and blue or black letters. [file Image_1.TIF]

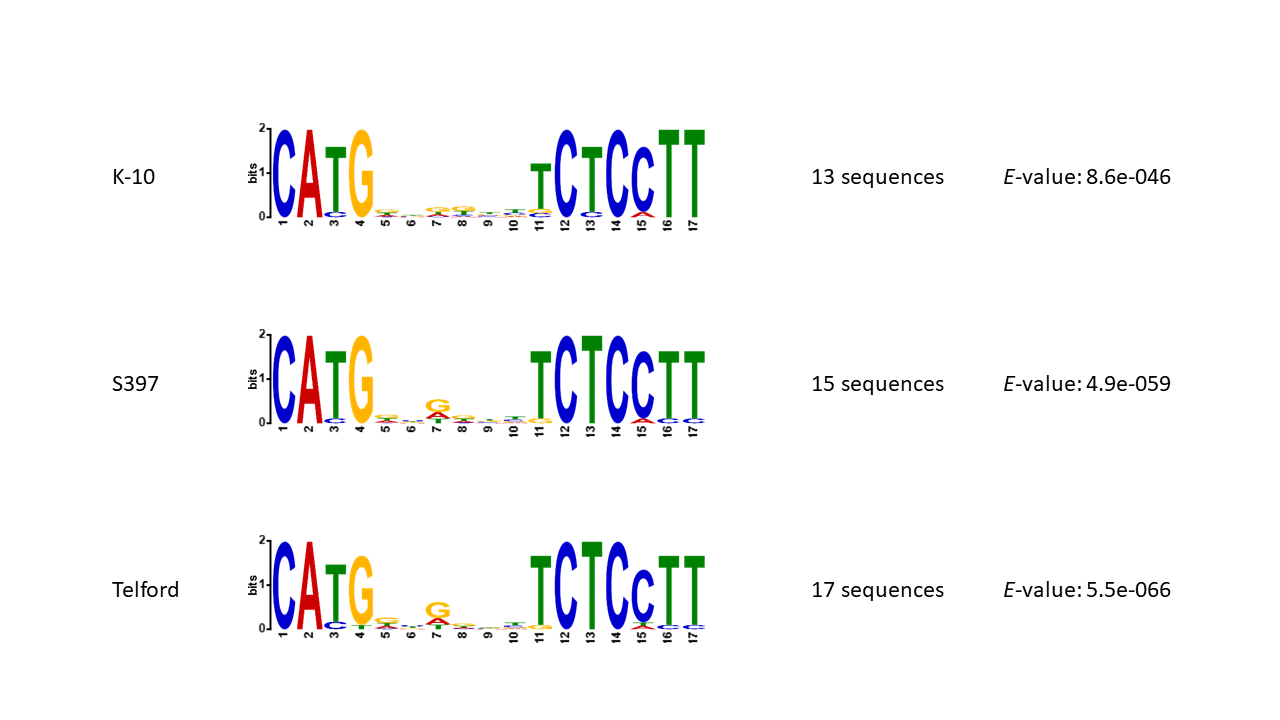

Supplement: Supplementary Figure 2 — MEME motif of putative IS900 insertion site extract from Mah 104 genome. Upstream and downstream region extracted from K-10, S397, and Telford genomes were aligned to the Mah 104 genome to find orthologous loci. Sequence of 14 to 17 bp present at conserved orthologous loci in the Mah 104 genome were extracted and MEME was used to discover conserved motif. [file Image_2.TIF]
