# Supplementary material for: Whole-Genome Analysis of Mycobacterium avium subsp. paratuberculosis IS900 Insertions Reveals Strain Type-Specific Modalities
Source: Front Microbiol. 2021 May 10;12:660002. doi: 10.3389/fmicb.2021.660002 (PMC8141618; doi:10.3389/fmicb.2021.660002)
Supplement: Supplementary Figure 3 — Detail of the genes surrounding all the IS900 loci. Representation of IS900 loci environment. Gene present 7500-bp upstream and downstream of each IS900 copies were considered. Gray box represents blastn IS900 hits. Presence of hed gene was indicated when a RBS was found upstream the gene (symbolized by a chevron). The first genes upstream and downstream IS900 copies were color filled according to their COG functional categories. Pseudo genes were indicated in black. [file Presentation_1.PPTX]

## Slide 1
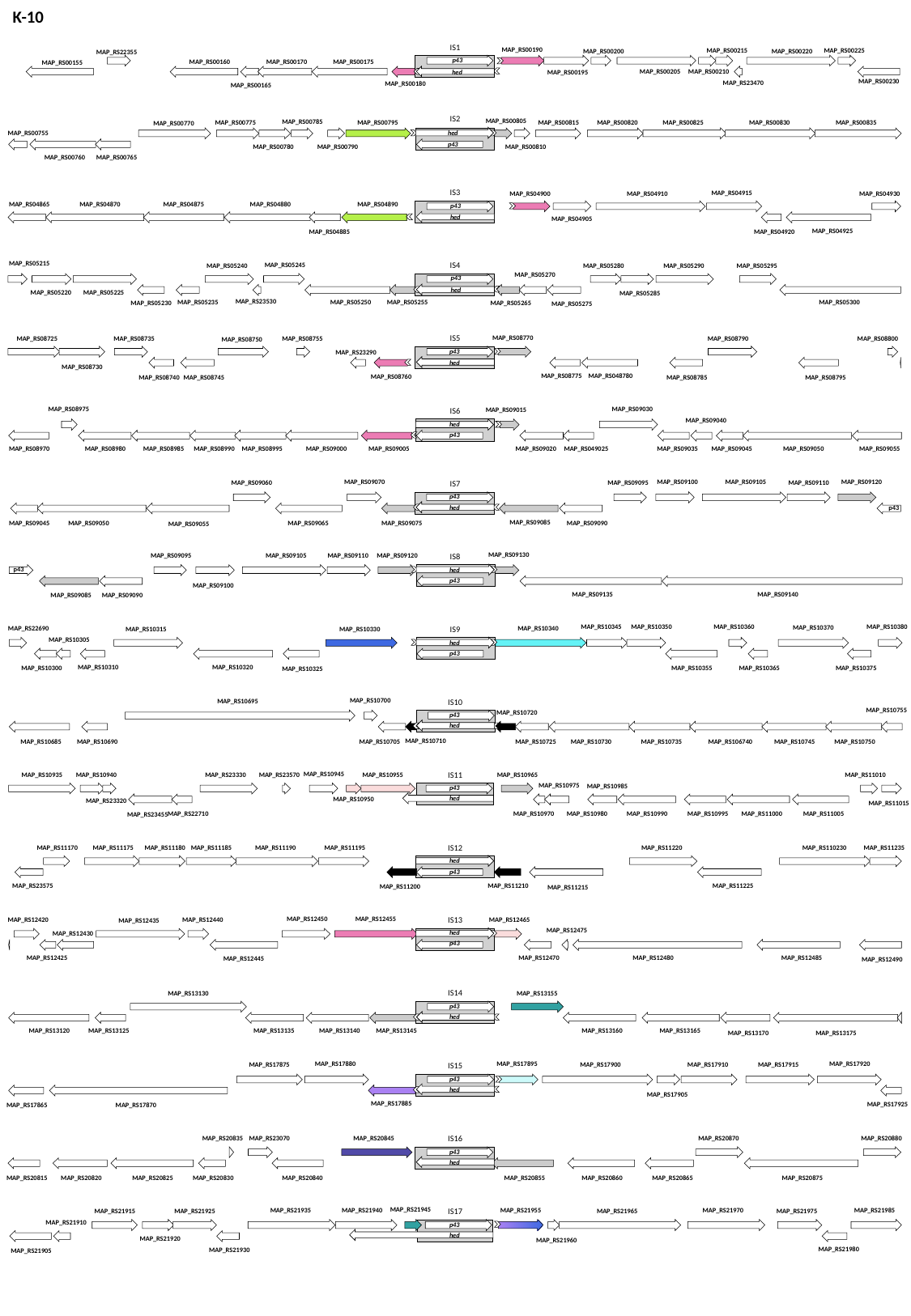

K-10
IS1
MAP_RS00190
MAP_RS00215
MAP_RS00225
MAP_RS00220
MAP_RS00200
MAP_RS22355
p43
hed
MAP_RS00175
MAP_RS00160
MAP_RS00170
MAP_RS00155
MAP_RS00205
MAP_RS00210
MAP_RS00195
MAP_RS00230
MAP_RS23470
MAP_RS00180
MAP_RS00165
IS2
MAP_RS00805
MAP_RS00785
MAP_RS00775
MAP_RS00815
MAP_RS00820
MAP_RS00825
MAP_RS00830
MAP_RS00835
MAP_RS00795
MAP_RS00770
MAP_RS00755
hed
p43
MAP_RS00780
MAP_RS00790
MAP_RS00810
MAP_RS00760
MAP_RS00765
IS3
MAP_RS04915
MAP_RS04900
MAP_RS04930
MAP_RS04910
MAP_RS04865
MAP_RS04870
MAP_RS04875
MAP_RS04890
MAP_RS04880
p43
hed
MAP_RS04905
MAP_RS04925
MAP_RS04885
MAP_RS04920
MAP_RS05215
IS4
MAP_RS05245
MAP_RS05295
MAP_RS05240
MAP_RS05290
MAP_RS05280
MAP_RS05270
p43
hed
MAP_RS05220
MAP_RS05225
MAP_RS05285
MAP_RS23530
MAP_RS05250
MAP_RS05300
MAP_RS05255
MAP_RS05235
MAP_RS05230
MAP_RS05265
MAP_RS05275
IS5
MAP_RS08770
MAP_RS08755
MAP_RS08725
MAP_RS08735
MAP_RS08790
MAP_RS08800
MAP_RS08750
p43
hed
MAP_RS23290
MAP_RS08730
MAP_RS08775
MAP_RS048780
MAP_RS08760
MAP_RS08740
MAP_RS08745
MAP_RS08785
MAP_RS08795
MAP_RS08975
MAP_RS09030
IS6
MAP_RS09015
MAP_RS09040
hed
p43
MAP_RS08970
MAP_RS08980
MAP_RS08985
MAP_RS08990
MAP_RS08995
MAP_RS049025
MAP_RS09035
MAP_RS09045
MAP_RS09050
MAP_RS09055
MAP_RS09000
MAP_RS09005
MAP_RS09020
MAP_RS09120
MAP_RS09100
MAP_RS09105
MAP_RS09070
MAP_RS09060
IS7
MAP_RS09095
MAP_RS09110
p43
hed
p43
MAP_RS09085
MAP_RS09075
MAP_RS09045
MAP_RS09050
MAP_RS09065
MAP_RS09090
MAP_RS09055
MAP_RS09130
MAP_RS09095
MAP_RS09105
MAP_RS09110
IS8
MAP_RS09120
p43
hed
p43
MAP_RS09100
MAP_RS09140
MAP_RS09135
MAP_RS09085
MAP_RS09090
MAP_RS10345
MAP_RS10350
MAP_RS10360
MAP_RS10380
MAP_RS10370
MAP_RS22690
IS9
MAP_RS10340
MAP_RS10315
MAP_RS10330
MAP_RS10305
hed
p43
MAP_RS10320
MAP_RS10310
MAP_RS10300
MAP_RS10365
MAP_RS10355
MAP_RS10375
MAP_RS10325
MAP_RS10700
MAP_RS10695
IS10
MAP_RS10755
MAP_RS10720
p43
hed
MAP_RS10710
MAP_RS10685
MAP_RS10690
MAP_RS10705
MAP_RS10725
MAP_RS10730
MAP_RS10735
MAP_RS106740
MAP_RS10745
MAP_RS10750
IS11
MAP_RS10945
MAP_RS23330
MAP_RS23570
MAP_RS10955
MAP_RS10935
MAP_RS10940
MAP_RS11010
MAP_RS10965
MAP_RS10975
MAP_RS10985
p43
hed
MAP_RS10950
MAP_RS23320
MAP_RS11015
MAP_RS22710
MAP_RS10970
MAP_RS10980
MAP_RS10990
MAP_RS10995
MAP_RS11000
MAP_RS11005
MAP_RS23455
IS12
MAP_RS11220
MAP_RS11175
MAP_RS11180
MAP_RS11185
MAP_RS11190
MAP_RS11195
MAP_RS11235
MAP_RS110230
MAP_RS11170
hed
p43
MAP_RS11225
MAP_RS23575
MAP_RS11210
MAP_RS11200
MAP_RS11215
MAP_RS12450
MAP_RS12455
IS13
MAP_RS12465
MAP_RS12420
MAP_RS12440
MAP_RS12435
MAP_RS12475
hed
p43
MAP_RS12430
MAP_RS12425
MAP_RS12470
MAP_RS12480
MAP_RS12485
MAP_RS12445
MAP_RS12490
IS14
MAP_RS13130
MAP_RS13155
p43
hed
MAP_RS13145
MAP_RS13135
MAP_RS13120
MAP_RS13125
MAP_RS13140
MAP_RS13160
MAP_RS13165
MAP_RS13170
MAP_RS13175
MAP_RS17880
MAP_RS17920
MAP_RS17895
MAP_RS17915
MAP_RS17875
IS15
MAP_RS17910
MAP_RS17900
p43
hed
MAP_RS17905
MAP_RS17885
MAP_RS17925
MAP_RS17865
MAP_RS17870
IS16
MAP_RS20835
MAP_RS23070
MAP_RS20845
MAP_RS20870
MAP_RS20880
p43
hed
MAP_RS20815
MAP_RS20820
MAP_RS20825
MAP_RS20830
MAP_RS20840
MAP_RS20855
MAP_RS20860
MAP_RS20865
MAP_RS20875
MAP_RS21945
MAP_RS21935
MAP_RS21940
IS17
MAP_RS21955
MAP_RS21970
MAP_RS21985
MAP_RS21915
MAP_RS21925
MAP_RS21965
MAP_RS21975
MAP_RS21910
p43
hed
MAP_RS21920
MAP_RS21960
MAP_RS21980
MAP_RS21930
MAP_RS21905

## Slide 2
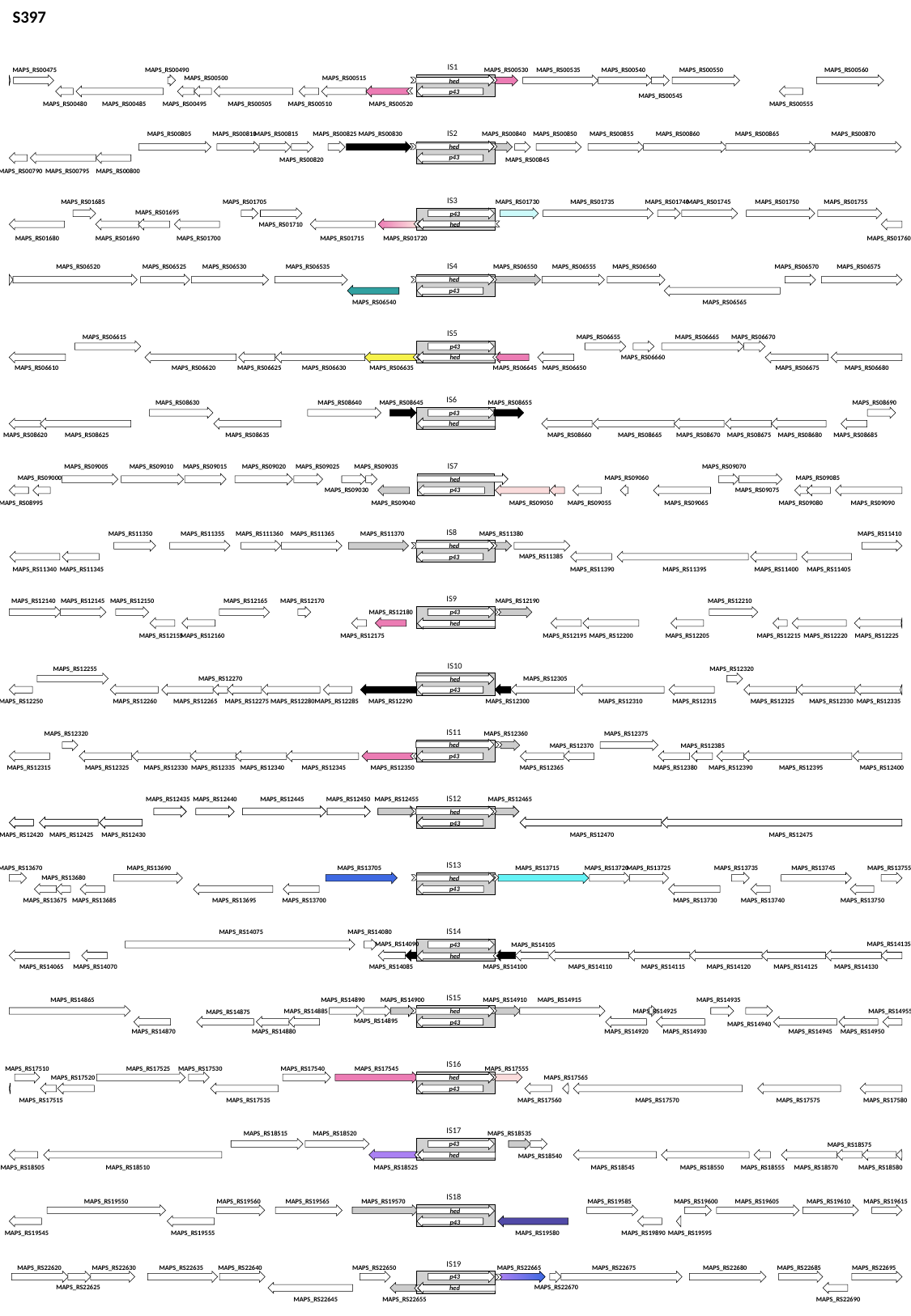

S397
IS1
MAPS_RS00475
MAPS_RS00490
MAPS_RS00530
MAPS_RS00535
MAPS_RS00540
MAPS_RS00550
MAPS_RS00560
MAPS_RS00500
MAPS_RS00515
hed
p43
MAPS_RS00545
MAPS_RS00480
MAPS_RS00485
MAPS_RS00495
MAPS_RS00505
MAPS_RS00510
MAPS_RS00520
MAPS_RS00555
IS2
MAPS_RS00805
MAPS_RS00810
MAPS_RS00815
MAPS_RS00825
MAPS_RS00830
MAPS_RS00840
MAPS_RS00850
MAPS_RS00855
MAPS_RS00860
MAPS_RS00865
MAPS_RS00870
hed
p43
MAPS_RS00820
MAPS_RS00845
MAPS_RS00790
MAPS_RS00795
MAPS_RS00800
IS3
MAPS_RS01685
MAPS_RS01705
MAPS_RS01730
MAPS_RS01735
MAPS_RS01740
MAPS_RS01745
MAPS_RS01750
MAPS_RS01755
MAPS_RS01695
p43
hed
MAPS_RS01710
MAPS_RS01720
MAPS_RS01680
MAPS_RS01690
MAPS_RS01700
MAPS_RS01715
MAPS_RS01760
IS4
MAPS_RS06520
MAPS_RS06525
MAPS_RS06530
MAPS_RS06535
MAPS_RS06550
MAPS_RS06555
MAPS_RS06560
MAPS_RS06570
MAPS_RS06575
hed
p43
MAPS_RS06540
MAPS_RS06565
IS5
MAPS_RS06615
MAPS_RS06655
MAPS_RS06665
MAPS_RS06670
p43
hed
MAPS_RS06660
MAPS_RS06610
MAPS_RS06620
MAPS_RS06625
MAPS_RS06630
MAPS_RS06635
MAPS_RS06645
MAPS_RS06650
MAPS_RS06675
MAPS_RS06680
IS6
MAPS_RS08630
MAPS_RS08640
MAPS_RS08645
MAPS_RS08655
MAPS_RS08690
p43
hed
MAPS_RS08620
MAPS_RS08625
MAPS_RS08635
MAPS_RS08660
MAPS_RS08665
MAPS_RS08670
MAPS_RS08675
MAPS_RS08680
MAPS_RS08685
IS7
MAPS_RS09025
MAPS_RS09005
MAPS_RS09010
MAPS_RS09015
MAPS_RS09020
MAPS_RS09035
MAPS_RS09070
MAPS_RS09060
MAPS_RS09085
MAPS_RS09000
hed
p43
MAPS_RS09030
MAPS_RS09075
MAPS_RS08995
MAPS_RS09040
MAPS_RS09050
MAPS_RS09055
MAPS_RS09065
MAPS_RS09080
MAPS_RS09090
IS8
MAPS_RS11350
MAPS_RS11355
MAPS_RS111360
MAPS_RS11365
MAPS_RS11370
MAPS_RS11380
MAPS_RS11410
hed
p43
MAPS_RS11385
MAPS_RS11340
MAPS_RS11345
MAPS_RS11390
MAPS_RS11395
MAPS_RS11400
MAPS_RS11405
IS9
MAPS_RS12140
MAPS_RS12145
MAPS_RS12150
MAPS_RS12165
MAPS_RS12170
MAPS_RS12190
MAPS_RS12210
MAPS_RS12180
p43
hed
MAPS_RS12155
MAPS_RS12160
MAPS_RS12175
MAPS_RS12195
MAPS_RS12200
MAPS_RS12205
MAPS_RS12215
MAPS_RS12220
MAPS_RS12225
IS10
MAPS_RS12255
MAPS_RS12320
MAPS_RS12270
MAPS_RS12305
hed
p43
MAPS_RS12250
MAPS_RS12260
MAPS_RS12265
MAPS_RS12275
MAPS_RS12280
MAPS_RS12285
MAPS_RS12290
MAPS_RS12300
MAPS_RS12310
MAPS_RS12315
MAPS_RS12325
MAPS_RS12330
MAPS_RS12335
IS11
MAPS_RS12320
MAPS_RS12360
MAPS_RS12375
hed
p43
MAPS_RS12385
MAPS_RS12370
MAPS_RS12315
MAPS_RS12325
MAPS_RS12330
MAPS_RS12335
MAPS_RS12340
MAPS_RS12345
MAPS_RS12350
MAPS_RS12365
MAPS_RS12380
MAPS_RS12390
MAPS_RS12395
MAPS_RS12400
IS12
MAPS_RS12435
MAPS_RS12440
MAPS_RS12445
MAPS_RS12450
MAPS_RS12455
MAPS_RS12465
hed
p43
MAPS_RS12420
MAPS_RS12425
MAPS_RS12430
MAPS_RS12470
MAPS_RS12475
IS13
MAPS_RS13670
MAPS_RS13690
MAPS_RS13705
MAPS_RS13715
MAPS_RS13720
MAPS_RS13725
MAPS_RS13735
MAPS_RS13745
MAPS_RS13755
MAPS_RS13680
hed
p43
MAPS_RS13675
MAPS_RS13685
MAPS_RS13695
MAPS_RS13700
MAPS_RS13730
MAPS_RS13740
MAPS_RS13750
IS14
MAPS_RS14080
MAPS_RS14075
MAPS_RS14135
MAPS_RS14090
MAPS_RS14105
p43
hed
MAPS_RS14065
MAPS_RS14070
MAPS_RS14085
MAPS_RS14100
MAPS_RS14110
MAPS_RS14115
MAPS_RS14120
MAPS_RS14125
MAPS_RS14130
IS15
MAPS_RS14865
MAPS_RS14890
MAPS_RS14900
MAPS_RS14910
MAPS_RS14915
MAPS_RS14935
MAPS_RS14885
MAPS_RS14925
MAPS_RS14955
hed
p43
MAPS_RS14875
MAPS_RS14895
MAPS_RS14940
MAPS_RS14870
MAPS_RS14880
MAPS_RS14920
MAPS_RS14930
MAPS_RS14945
MAPS_RS14950
IS16
MAPS_RS17510
MAPS_RS17525
MAPS_RS17530
MAPS_RS17540
MAPS_RS17545
MAPS_RS17555
MAPS_RS17520
hed
p43
MAPS_RS17565
MAPS_RS17515
MAPS_RS17535
MAPS_RS17560
MAPS_RS17570
MAPS_RS17575
MAPS_RS17580
IS17
MAPS_RS18515
MAPS_RS18520
MAPS_RS18535
p43
hed
MAPS_RS18575
MAPS_RS18540
MAPS_RS18505
MAPS_RS18510
MAPS_RS18525
MAPS_RS18545
MAPS_RS18550
MAPS_RS18555
MAPS_RS18570
MAPS_RS18580
IS18
MAPS_RS19550
MAPS_RS19560
MAPS_RS19565
MAPS_RS19570
MAPS_RS19585
MAPS_RS19600
MAPS_RS19605
MAPS_RS19610
MAPS_RS19615
hed
p43
MAPS_RS19545
MAPS_RS19555
MAPS_RS19580
MAPS_RS19890
MAPS_RS19595
IS19
MAPS_RS22620
MAPS_RS22630
MAPS_RS22635
MAPS_RS22640
MAPS_RS22650
MAPS_RS22665
MAPS_RS22675
MAPS_RS22680
MAPS_RS22685
MAPS_RS22695
p43
hed
MAPS_RS22625
MAPS_RS22670
MAPS_RS22645
MAPS_RS22655
MAPS_RS22690

## Slide 3
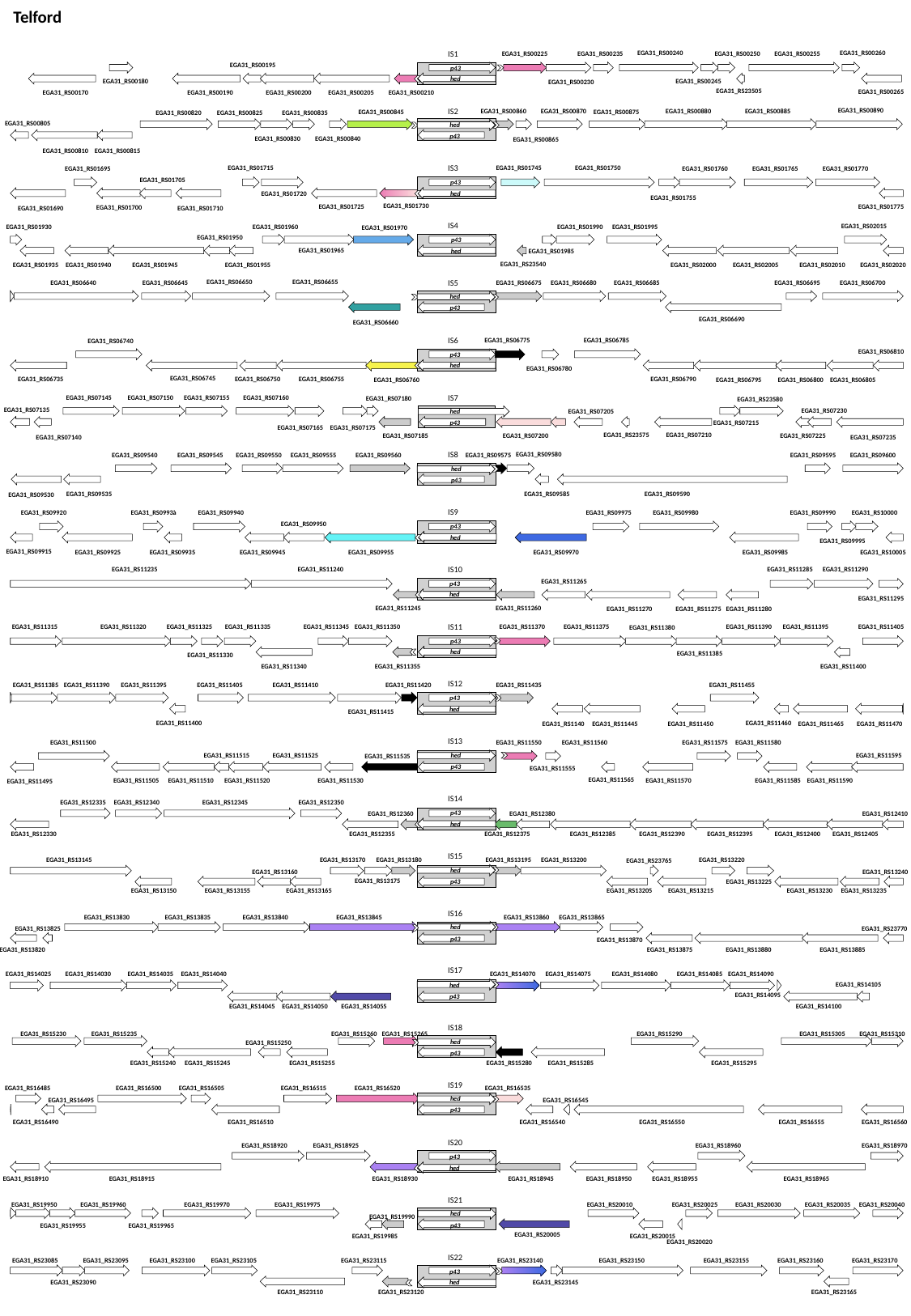

Telford
IS1
EGA31_RS00240
EGA31_RS00260
EGA31_RS00235
EGA31_RS00225
EGA31_RS00255
EGA31_RS00250
EGA31_RS00195
p43
hed
EGA31_RS00180
EGA31_RS00245
EGA31_RS00230
EGA31_RS23505
EGA31_RS00265
EGA31_RS00170
EGA31_RS00190
EGA31_RS00200
EGA31_RS00205
EGA31_RS00210
IS2
EGA31_RS00890
EGA31_RS00860
EGA31_RS00880
EGA31_RS00885
EGA31_RS00870
EGA31_RS00875
EGA31_RS00845
EGA31_RS00820
EGA31_RS00825
EGA31_RS00835
EGA31_RS00805
hed
p43
EGA31_RS00830
EGA31_RS00840
EGA31_RS00865
EGA31_RS00810
EGA31_RS00815
IS3
EGA31_RS01745
EGA31_RS01750
EGA31_RS01715
EGA31_RS01695
EGA31_RS01760
EGA31_RS01765
EGA31_RS01770
EGA31_RS01705
p43
hed
EGA31_RS01720
EGA31_RS01755
EGA31_RS01730
EGA31_RS01775
EGA31_RS01725
EGA31_RS01700
EGA31_RS01690
EGA31_RS01710
IS4
EGA31_RS02015
EGA31_RS01995
EGA31_RS01930
EGA31_RS01990
EGA31_RS01960
EGA31_RS01970
EGA31_RS01950
p43
hed
EGA31_RS01965
EGA31_RS01985
EGA31_RS23540
EGA31_RS01935
EGA31_RS02005
EGA31_RS02000
EGA31_RS02010
EGA31_RS02020
EGA31_RS01940
EGA31_RS01955
EGA31_RS01945
IS5
EGA31_RS06650
EGA31_RS06655
EGA31_RS06675
EGA31_RS06640
EGA31_RS06685
EGA31_RS06695
EGA31_RS06700
EGA31_RS06645
EGA31_RS06680
hed
p43
EGA31_RS06690
EGA31_RS06660
IS6
EGA31_RS06775
EGA31_RS06785
EGA31_RS06740
EGA31_RS06810
p43
hed
EGA31_RS06780
EGA31_RS06745
EGA31_RS06735
EGA31_RS06750
EGA31_RS06755
EGA31_RS06790
EGA31_RS06805
EGA31_RS06800
EGA31_RS06760
EGA31_RS06795
IS7
EGA31_RS07145
EGA31_RS07150
EGA31_RS07155
EGA31_RS07160
EGA31_RS07180
EGA31_RS23580
EGA31_RS07135
EGA31_RS07230
EGA31_RS07205
hed
p43
EGA31_RS07215
EGA31_RS07165
EGA31_RS07175
EGA31_RS23575
EGA31_RS07210
EGA31_RS07200
EGA31_RS07225
EGA31_RS07185
EGA31_RS07235
EGA31_RS07140
IS8
EGA31_RS09580
EGA31_RS09575
EGA31_RS09540
EGA31_RS09545
EGA31_RS09550
EGA31_RS09560
EGA31_RS09595
EGA31_RS09600
EGA31_RS09555
hed
p43
EGA31_RS09535
EGA31_RS09585
EGA31_RS09590
EGA31_RS09530
IS9
EGA31_RS09920
EGA31_RS0993à
EGA31_RS09940
EGA31_RS09975
EGA31_RS09980
EGA31_RS09990
EGA31_RS10000
EGA31_RS09950
p43
hed
EGA31_RS09995
EGA31_RS09915
EGA31_RS09935
EGA31_RS09955
EGA31_RS09925
EGA31_RS09945
EGA31_RS09970
EGA31_RS10005
EGA31_RS09985
IS10
EGA31_RS11235
EGA31_RS11240
EGA31_RS11285
EGA31_RS11290
EGA31_RS11265
p43
hed
EGA31_RS11295
EGA31_RS11245
EGA31_RS11260
EGA31_RS11275
EGA31_RS11270
EGA31_RS11280
IS11
EGA31_RS11350
EGA31_RS11315
EGA31_RS11320
EGA31_RS11335
EGA31_RS11345
EGA31_RS11375
EGA31_RS11390
EGA31_RS11395
EGA31_RS11405
EGA31_RS11370
EGA31_RS11325
EGA31_RS11380
p43
hed
EGA31_RS11385
EGA31_RS11330
EGA31_RS11355
EGA31_RS11340
EGA31_RS11400
IS12
EGA31_RS11435
EGA31_RS11420
EGA31_RS11385
EGA31_RS11390
EGA31_RS11395
EGA31_RS11405
EGA31_RS11410
EGA31_RS11455
p43
hed
EGA31_RS11415
EGA31_RS11400
EGA31_RS11460
EGA31_RS1140
EGA31_RS11445
EGA31_RS11450
EGA31_RS11465
EGA31_RS11470
IS13
EGA31_RS11500
EGA31_RS11560
EGA31_RS11575
EGA31_RS11580
EGA31_RS11550
EGA31_RS11515
EGA31_RS11525
EGA31_RS11595
hed
p43
EGA31_RS11535
EGA31_RS11555
EGA31_RS11565
EGA31_RS11505
EGA31_RS11510
EGA31_RS11520
EGA31_RS11570
EGA31_RS11585
EGA31_RS11590
EGA31_RS11530
EGA31_RS11495
IS14
EGA31_RS12335
EGA31_RS12340
EGA31_RS12345
EGA31_RS12350
p43
hed
EGA31_RS12360
EGA31_RS12380
EGA31_RS12410
EGA31_RS12330
EGA31_RS12355
EGA31_RS12375
EGA31_RS12385
EGA31_RS12390
EGA31_RS12395
EGA31_RS12400
EGA31_RS12405
IS15
EGA31_RS13145
EGA31_RS13170
EGA31_RS13180
EGA31_RS13195
EGA31_RS13200
EGA31_RS13220
EGA31_RS23765
hed
p43
EGA31_RS13160
EGA31_RS13240
EGA31_RS13175
EGA31_RS13225
EGA31_RS13150
EGA31_RS13155
EGA31_RS13165
EGA31_RS13205
EGA31_RS13215
EGA31_RS13230
EGA31_RS13235
IS16
EGA31_RS13830
EGA31_RS13835
EGA31_RS13840
EGA31_RS13845
EGA31_RS13860
EGA31_RS13865
hed
p43
EGA31_RS13825
EGA31_RS23770
EGA31_RS13870
EGA31_RS13820
EGA31_RS13875
EGA31_RS13880
EGA31_RS13885
IS17
EGA31_RS14025
EGA31_RS14030
EGA31_RS14035
EGA31_RS14040
EGA31_RS14070
EGA31_RS14075
EGA31_RS14080
EGA31_RS14085
EGA31_RS14090
EGA31_RS14105
hed
p43
EGA31_RS14095
EGA31_RS14045
EGA31_RS14050
EGA31_RS14055
EGA31_RS14100
IS18
EGA31_RS15230
EGA31_RS15235
EGA31_RS15260
EGA31_RS15265
EGA31_RS15290
EGA31_RS15305
EGA31_RS15310
hed
p43
EGA31_RS15250
EGA31_RS15240
EGA31_RS15245
EGA31_RS15255
EGA31_RS15280
EGA31_RS15285
EGA31_RS15295
IS19
EGA31_RS16485
EGA31_RS16500
EGA31_RS16505
EGA31_RS16515
EGA31_RS16520
EGA31_RS16535
hed
p43
EGA31_RS16495
EGA31_RS16545
EGA31_RS16490
EGA31_RS16510
EGA31_RS16540
EGA31_RS16550
EGA31_RS16555
EGA31_RS16560
IS20
EGA31_RS18920
EGA31_RS18925
EGA31_RS18960
EGA31_RS18970
p43
hed
EGA31_RS18910
EGA31_RS18915
EGA31_RS18930
EGA31_RS18945
EGA31_RS18950
EGA31_RS18955
EGA31_RS18965
IS21
EGA31_RS19950
EGA31_RS19960
EGA31_RS19970
EGA31_RS19975
EGA31_RS20010
EGA31_RS20025
EGA31_RS20030
EGA31_RS20035
EGA31_RS20040
hed
p43
EGA31_RS19990
EGA31_RS19955
EGA31_RS19965
EGA31_RS20005
EGA31_RS20015
EGA31_RS19985
EGA31_RS20020
IS22
EGA31_RS23085
EGA31_RS23095
EGA31_RS23100
EGA31_RS23105
EGA31_RS23115
EGA31_RS23140
EGA31_RS23150
EGA31_RS23155
EGA31_RS23160
EGA31_RS23170
p43
hed
EGA31_RS23090
EGA31_RS23145
EGA31_RS23110
EGA31_RS23120
EGA31_RS23165

## Slide 4
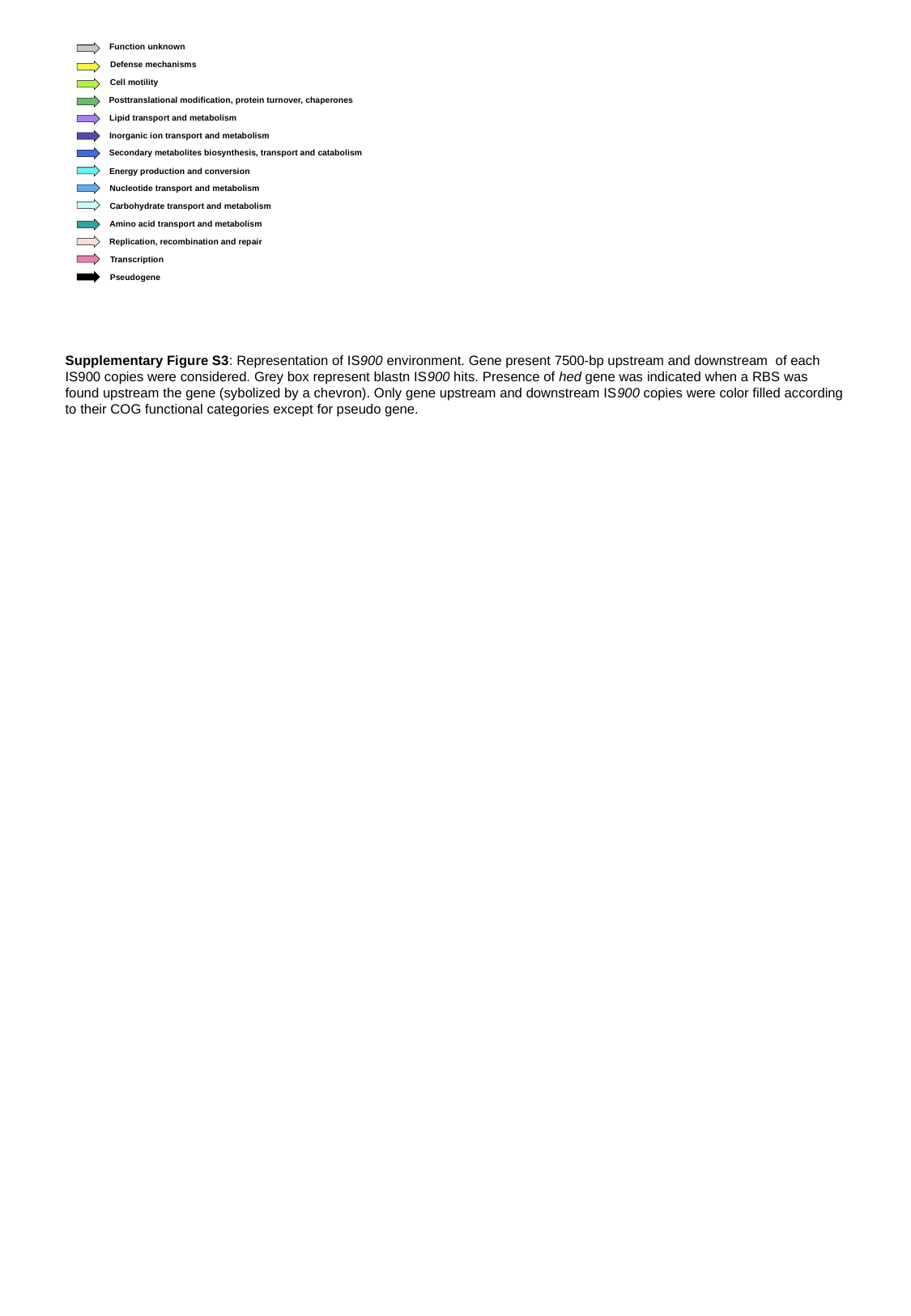

Function unknown
Defense mechanisms
Cell motility
Posttranslational modification, protein turnover, chaperones
Lipid transport and metabolism
Inorganic ion transport and metabolism
Secondary metabolites biosynthesis, transport and catabolism
Energy production and conversion
Nucleotide transport and metabolism
Carbohydrate transport and metabolism
Amino acid transport and metabolism
Replication, recombination and repair
Transcription
Pseudogene
Supplementary Figure S3: Representation of IS900 environment. Gene present 7500-bp upstream and downstream of each IS900 copies were considered. Grey box represent blastn IS900 hits. Presence of hed gene was indicated when a RBS was found upstream the gene (sybolized by a chevron). Only gene upstream and downstream IS900 copies were color filled according to their COG functional categories except for pseudo gene.
